# Effects of described demonstrator ability on brain and behavior when learning from others

**DOI:** 10.1038/s41539-024-00292-0

**Published:** 2025-01-16

**Authors:** Ida Selbing, Nina Becker, Yafeng Pan, Björn Lindström, Andreas Olsson

**Affiliations:** 1 Division of Psychology, Karolinska Institutet, Solna, Sweden; 2 Department of Psychology and Behavioral Sciences, Zhejiang University, Hangzhou, China

**Keywords:** Cognitive neuroscience, Human behaviour

## Abstract

Observational learning enables us to make decisions by watching others’ behaviors. The quality of such learning depends on the abilities of those we observe, but also on our beliefs about those abilities. We have previously demonstrated that observers learned better from demonstrators described as high vs. low in ability, regardless of their actual performance. The current study aimed to conceptually replicate these findings, and explore the neural mechanisms involved. Forty-five participants performed an observational learning task while undergoing functional magnetic resonance imaging (fMRI). We hypothesized that participants would perform better when demonstrators were described as having high vs. low ability. Unexpectedly, participants performed equally well regardless of described demonstrator ability. The behavioral effects of biased observational learning seem to be driven by mentalizing processes together with general learning and decision-making processes.

## Introduction

Social learning, the ability to learn from others, is essential for both humans and non-human animals and enables accumulation of knowledge and culture^1–3^. Importantly, the quality of such learning depends on whom we learn from^4^. Indeed, previous research has shown that social learning is influenced by perceived competence, skill and knowledge of the individual we learn from, the so called “demonstrator”^5–7^. Yet, little is known about the mechanisms underlying the impact of social impressions on social learning. Here, we leveraged brain imaging and computational methods to study how observational learning of a simple avoidance task was affected by described demonstrator ability.

Learning about others’ actions through observation is commonly divided into two forms of learning: 1) learning by observing the action of the demonstrator, and 2) learning by observing the outcome of a demonstrator’s actions. For simplicity, we will refer to the former as “copying”, even though the underlying learning mechanism might differ from simple imitation or decision biasing^8^. Learning from observation of the outcome of a demonstrator’s actions is typically thought of as learning to predict (the value of) the outcome of actions through observation^9,10^, and will here be referred to as “observational outcome learning”.

Copying is an efficient form of learning when the demonstrator makes better choices than you, i.e., has a higher ability^4,11^. In order to identify who has high ability, social learning strategies such as “copy from the successful” can function as valuable heuristics. For observational outcome learning, there is a less straightforward link between the ability of the demonstrator and the efficiency of learning, because observers can also learn from the outcomes of poor decisions. In some cases, learning from someone with low ability making poor decisions might even be more efficient than learning from someone with high ability since this might allow you to explore different actions vicariously, through observation (Selbing, in prep.).

Ability-biased observational learning can thus be efficient during copying but less clearly so during observational outcome learning. When both the action of a demonstrator and the outcome of that action is available to an observer, both forms of learning are possible. In a previous study^12^, we investigated how beliefs about demonstrator ability and actual ability affected observational learning in a task which allowed both copying and observational outcome learning. We showed that observers performed better following observation of a demonstrator described as high vs. low in ability, and that this effect seemed to be driven by differences in observational outcome learning based on described ability. Actual ability had no effect on performance. Through analyzes of changes in pupil size, we suggested that the effect of described ability on performance could be explained by increased attention directed towards the behaviors and outcomes of the demonstrator believed to be high in ability, leading to more efficient learning from such demonstrators. This explanation is consistent with theories suggesting that humans often tend to exhibit success-biased attention^13,14^. One reason for such bias could be that there are many situations where it would be more valuable to pay attention to someone with high, rather than low, ability, e.g., when copying^4^.

Against this background, the current study had two main goals. First, we aimed to conceptually replicate the main results from our previous work described above^12^. Thus, based on our previous findings, we hypothesized that observational learning would be more efficient, and performance would be higher, when the demonstrator was described as having a high rather than low ability. Second, we wanted to investigate the neural correlates of this biased observational learning to get a better understanding of the underlying mechanisms. We expected that the learning bias would be linked to brain activity known to support attentional processes and social cognition.

To address these aims, participants performed a probabilistic two-choice task similar, but not identical, to the one used in our previous study^12^ while undergoing functional magnetic resonance imaging (fMRI). Since we were interested specifically in described, but not actual, ability as in the previous study, demonstrators were described as having either a high or a low ability, although demonstrators’ actual ability were pre-programmed to be low throughout the experiment. The task was also adapted for collection of fMRI data. We expected that participants would perform better when the demonstrators were described as having a high as compared to low ability. Participant’s behavior was analyzed using both standard statistical methods and reinforcement learning (RL) modeling^15^. The use of RL modeling allowed us to formalize the mechanisms of observational learning and get a more detailed understanding of the effects of described demonstrator ability, and crucially, relate these mechanisms to the neural data^16^.

At the neural level, we focused on regions associated with observational learning, social impression formation and mentalizing. Specifically, we examined areas known to play a role in observational learning and other related aspects of social cognition include the dorsolateral and dorsomedial prefrontal cortex (dlPFC and dmPFC), the ventromedial prefrontal cortex (vmPFC), and the amygdala^9,10,17–22^. Additionally, we considered the temporoparietal regions and the PCC, which are typically associated with mentalizing processes, but are also relevant for social learning and susceptibility to social influence^23–26^. Our investigation aimed to determine whether activity in these regions varied based on the described ability of the demonstrator.

## Results

### Demonstrator descriptions affect ratings of their performance

**Fig. 1:**
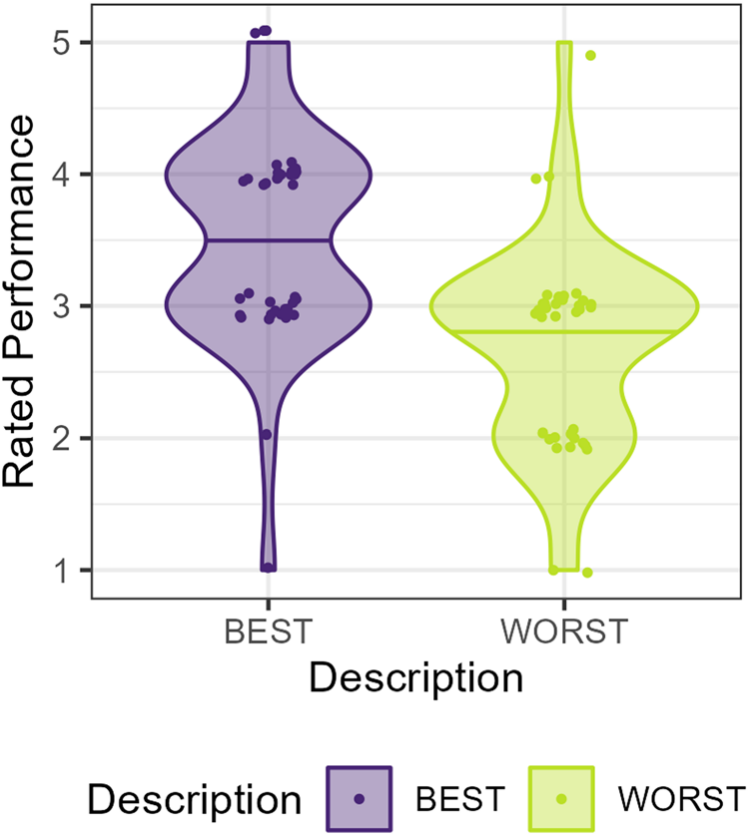
Demonstrator performance rating as a function of demonstrator description. Performance was rated from one (very poor) to five (very good). Data are represented with two data points per participant. Data points are slightly jittered for illustrative purposes.

First, as a manipulation check, we tested if the ratings of the performance of the observed demonstrators were affected by demonstrator description. Indeed, demonstrators described as Best vs. Worst were rated as performing better (*β* *=* 2.32, *SE* *=* 0.57, *Z* *=* 4.10, *p* ≤ 0.001), Fig. 1. Likelihood ratio tests showed no improvement in model fit by including either a direct measure of demonstrator performance (proportion of optimal choices) as predictor (*p* *=* 0.094) nor an indirect measure (proportion of trials were the demonstrator did not receive a shock) (*p* *=* 0.13). This demonstrates that a simple description of demonstrator ability indeed did influence the participants’ beliefs about the demonstrators, see Fig. 1.

### Demonstrator descriptions affect participants’ behavior

We first tested our prediction that participants would perform better during observation of demonstrators described as Best, compared to Worst, using a GLMM, where the dependent variable was the participants’ choices coded according to the choice optimality (Optimal, Suboptimal). As predictors, we included Description and Trial. Our analysis showed a main effect of Trial (*p* *=* 0.002), indicative of learning over time but, contrary to our predictions, no significant effect of Description on choice optimality (*p* *=* 0.996).

**Fig. 2:**
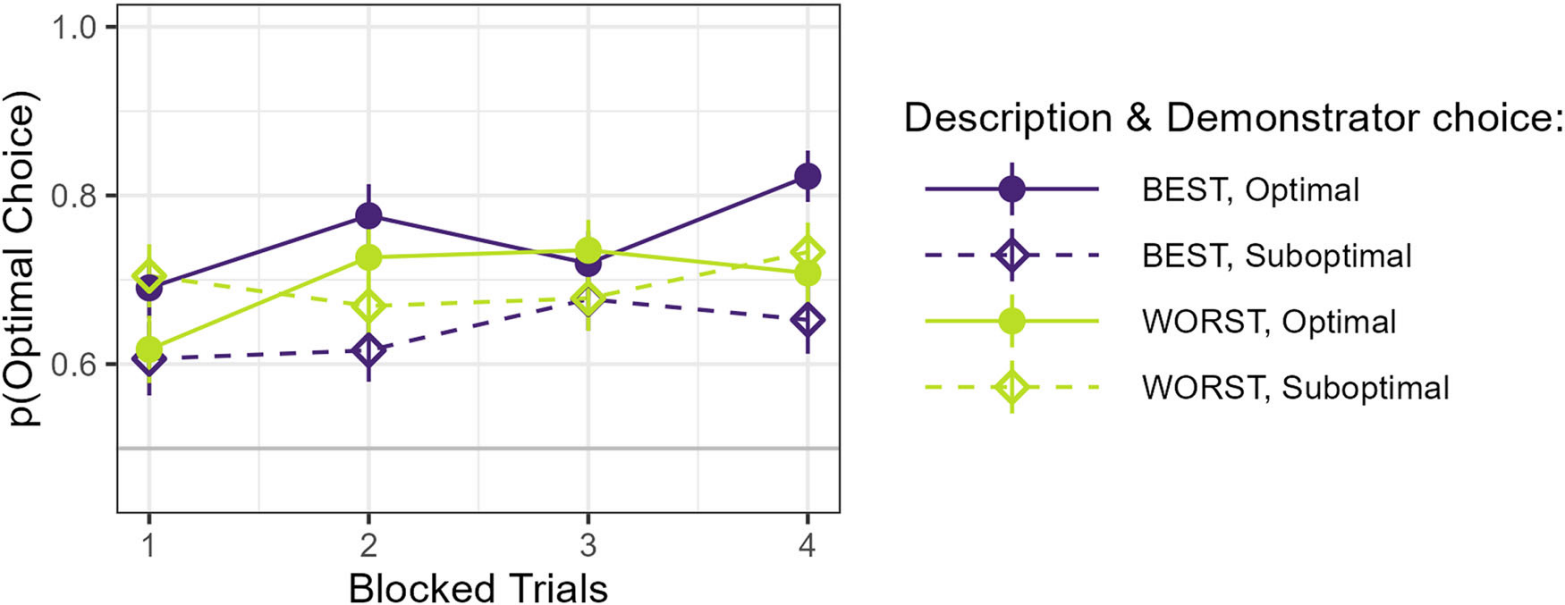
Demonstrator performance. Performance plotted as a function of Trial (blocked pairwise for clarity), Description, and Demonstrator choice.

Since these results differ from the findings in our earlier study^12^, we wanted to understand what could be driving them. Therefore, we then performed a set of post-hoc analyses to test whether participants’ behavior could have been affected by a tendency to copy choices made by the observed demonstrator. We started by adding the Demonstrator’s choice as a predictor to our model, coded according to optimality (Optimal, Suboptimal) to test if there was a general effect of copying. Although this significantly improved the model fit (*p* *<* 0.001) our analysis showed no effect of Demonstrator’s choice (*p* = 0.18). Next, we investigated if copying was sensitive to described ability by including the interaction between Description and Demonstrator’s choice in our model. This further improved the model fit (*p* *=* 0.025). Again, there was a significant main effect of Trial (*β* *=* 0.93, SE *=* 0.32, *Z* *=* 2.93, *p* *=* 0.003), indicating that participants learned over time, but no main effects of either Description (*p* *=* 0.943) or Demonstrator choice (*p* *=* 0.188). Most importantly, there was an interaction between Description and Demonstrator choice (*β* *=* 0.71, SE = 0.22, *Z* *=* 3.30, *p* *<* 0.001) caused by a higher tendency to select the same choice as the demonstrator following observation of a demonstrator described as Best compared to Worst, see Fig. 2. This indicates that participants copied the choices of the demonstrator to a larger extent (or possibly avoided to a lower extent) when the demonstrator was described as Best compared to Worst. Next, we investigated whether this effect changed over time by testing the three-way interaction between Description, Demonstrator choice, and Block. Such a change could for instance be driven by a tendency to learn not to copy over time (since copying here leads to poor performance). Our analysis did not show any such interaction with Block (*p* *=* 0.61). Participants thus did not appear to change their behavior systematically over the course of the experiment.

### RL modeling results

RL modeling was applied to get a more detailed understanding of participants’ behavior and to extract latent variables of learning to be used in the fMRI analyses. First, we identified what model best-described participants’ behavior, specifically focusing on which form of observational learning they used and if observational learning was sensitive to the described ability. Then, for models of interest, which included description-sensitive observational learning, we analyzed the fitted parameters to understand the nature of this sensitivity.

Analyzes of the RL models that best fitted each participant’s behavior were used to investigate how participants learned from the demonstrator. First, we set out to identify the model that best described the behavior of all participants, i.e., the model with the lowest mean AIC over all participants. This was a model which included both copying and observational outcome learning and where the copying rate was sensitive to the demonstrators’ description. Specifically, copying and/or avoidance was included during observation of the Worst, but not the Best, demonstrators (see Supplementary Information and below for details). In line with our behavioral analyses, the model indicates that participants appear to copy observed choices to a larger degree than we had expected^12,27^, and that described ability affected copying rather than observational outcome learning.

However, based on the rule of thumb suggested by Burnham and Andersson^28^, this best model has substantial support (a difference in AIC of at least 2) only when compared to the 26th best model or worse and thus does not stand out as a clear winner. We therefore decided to investigate if we could identify any patterns in the models that best described each individual participant. All but one participant was best described with a model that included one or both forms of observational learning: one where behavior was best described by a model of pure observational outcome learning (17 participants), and one that included both forms of observational learning, i.e., mixed strategy learning (19 participants). Thus, roughly half of the participants had some tendency to copy (or avoid) the demonstrator’s choice. It is important to note that given that demonstrators behaved randomly, copying was never an efficient learning strategy. Based on previous research, we had not expected participants to copy to such extent given that the outcomes of the demonstrator’s choices were available^12,27^.

To investigate if the subgroups learned from observation differently as a function of demonstrator description, we examined the models which best described each of the two main subgroups of participants identified above. The model which best described the behavior of the observational outcome learners included descriptive-sensitive observational outcome learning $({\alpha }_{{Observational}}^{{Best}}\ne {\alpha }_{{Observational}}^{{Worst}})$. The model which best described the mixed strategy learners instead included descriptive-sensitive copying $({\kappa }^{{Best}}\ne {\kappa }^{{Worst}})$ whereas observational outcome learning was descriptive-insensitive. Thus, participants could best be described as being descriptive-sensitive either during learning from observed outcomes, or during copying.

**Fig. 3:**
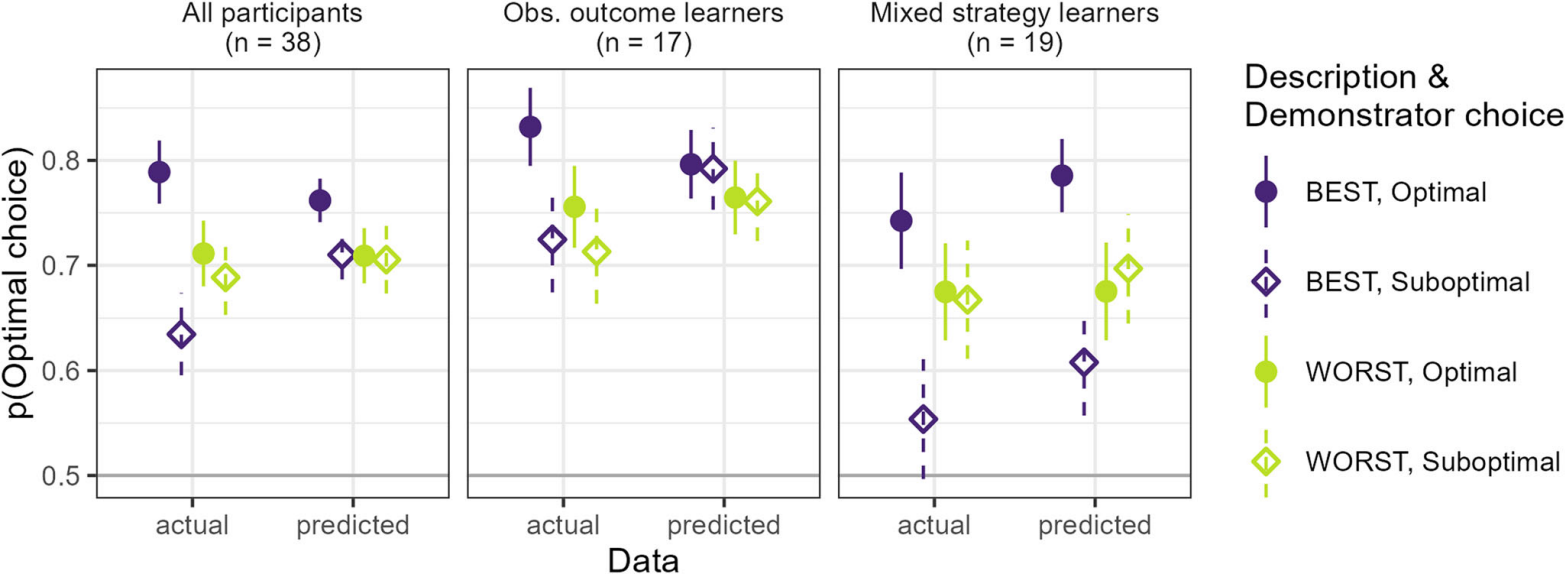
Model predictions. Model-based predicted mean behavior plotted against actual mean behavior for the three RL models that best describe either all participants or the two main subgroups of participants best described by either observational outcome learning models or mixed strategy learning models. The difference in performance depending on which choice the demonstrator made, appears to be driven mainly by the subgroup of mixed-strategy learners. Error bars indicate standard error of the mean.

We also evaluated how well these three models (the best model overall and the best model for each of the two subgroups of participants) were able to capture participants’ behavior. This was done by calculating the mean probability of the data, over all participants and trials, for each model. All models could predict participants’ behavior to 70% or more (the best overall model: 70%, best model for subgroup of observational outcome learners: 76%, best model for subgroup of mixed strategy learners: 70%). The best overall model qualitatively captured the pattern of participants’ behavior rather well apart from the effect of the interaction between demonstrator description and optimality of the demonstrator’s choice, see Fig. 3, leftmost panel. This interaction effect appears instead to be driven by those individuals whose behavior is best described by models that include copying (mixed strategy models), see Fig. 3. For a more detailed overview of how well the models captures behavior over time, see Supplementary Information.

**Fig. 4:**
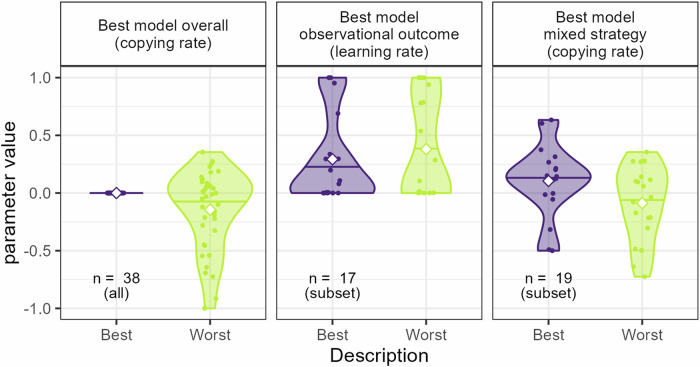
Fitted parameters. Parameter values of the fitted descriptive-sensitive parameters for the models best describe the overall data or data from the two main subgroups of participants. All these models included some form of description-sensitive learning. The white squares indicate mean parameter value. Note that the copying rate for the Best demonstrators in the best model overall (leftmost panel) is fixed at 0.

To get a more detailed understanding of how the described ability affected learning, we analyzed the estimated observational learning parameters for the three best models mentioned above. More specifically, for those cases where the models included descriptive-sensitive learning, i.e., where different learning parameters were fitted for the Best and Worst demonstrators respectively, relevant parameters were analyzed to get a better understanding of that effect, see also Fig. 4. For the overall best model, which included description-sensitive copying (where *κ*^*Best*^ *=* 0 and *κ*^*Worst*^ was a free parameter), the analysis showed that *κ*^*Worst*^ significantly differed from zero (*t*(37) = −2.62, *p* *=* 0.013), the mean value of *κ*^*Worst*^ was −0.14 and the median was −0.02. This indicated an overall tendency to avoid choices made by the demonstrators described as Worst. An analysis of the best model for the subgroup of pure observational outcome learners showed no systematic difference in observational learning rates as a function of demonstrator description (*Mean* *=* −0.09, *t*(16.00) = −0.7, *p* *=* 0.49). An analysis of the best model for the subgroup of mixed strategy learners showed a significant difference in copying rates as a function of demonstrator description (*t* (18) = 2.57, *p* *=* 0.019), with a mean difference of 0.19, indicating higher copying (or less avoidance) of choices made by demonstrators described as Best. An analysis of the separate copying rates showed that although mean *κ*^*Best*^ was positive (Mean = 0.1), and mean *κ*^*Worst*^ was slightly negative (Mean = −0.09), none of the parameters were significantly separate from zero (minimum *p* *=* 0.15). Thus, in line with our behavioral findings, demonstrator description seemed to influence observational learning by affecting the tendencies to copy demonstrators, such that the participants copied the choices of Best demonstrators to a larger extent than the Worst demonstrators.

### Imaging results

All brain regions showing significant activity are presented in Table 1. In the analyses, of both the observation phase and the action phase, we also performed Best > Worst and Worst > Best contrasts. These did not yield any significant results.

**Table 1:** Significant activity

*Region*	*BA*	*k*	*t*	*x*	*y*	*z*
Observational phase						
Contrast: Demonstrator choice (Worst) > baseline						
Temporal Mid R	37	1371	11.46	46	−64	2
Temporal Mid L	37	1194	9.07	-52	−68	6
SupraMarginal R	40	110	6.60	38	−34	44
Contrast: Demonstrator choice (Best) > baseline						
Temporal Mid R	37	1172	9.95	48	−60	4
Temporal Mid L	37	1087	8.33	−50	−68	6
Contrast: Demonstrator outcome (Worst) > baseline						
Temporal Mid R	37	3024	13.79	48	−64	2
Temporal Mid L	37	2350	12.39	−50	−68	6
Frontal Sup Medial R	32	287	6.77	8	26	44
Frontal Inf Tri R	45	1032	6.52	50	32	30
Contrast: Demonstrator outcome (Best) > baseline						
Temporal Mid R	37	2431	10.35	46	−64	2
Occipital Mid L	37	1909	8.78	−48	−68	6
Frontal Inf Tri R	45	954	7.96	44	28	26
Frontal Sup Medial L	32	354	7.00	−4	20	42
Action phase						
Contrast: Presentation of stimuli (Worst) > baseline						
Temporal Mid L	21	86	4.53	−56	−4	−18
Presentation of stimuli (Best) modulated by observer’s copying behavior						
SupraMarginal R	40	1262	10.68	62	−24	24
SupraMarginal L	40	1432	8.89	−60	−26	24
Temporal Mid L	19	282	8.12	−52	−70	4
Cingulum Mid R	8	256	8.07	2	24	38
Temporal Inf R	37	530	7.48	50	−70	−2

Brain regions showing significant activity during the observational and action phase.

*k* number of voxels, *BA* Brodmann area.

Our analysis of brain activity during the observation phase revealed changes in brain responses at the presentation of the demonstrator’s choice and the outcome (see Table 1). For the presentation of the demonstrator’s choice, there was an increase in activity in the middle temporal cortex for demonstrators described as both Best and Worst as compared to baseline. A paired-sample t-test revealed that there were, however, no significant differences as a function of demonstrator description. During the presentation of the outcome, an increase in activity was identified in the middle temporal cortex, medial, and inferior superior frontal cortex, again for demonstrators described as both Best and Worst as compared to baseline. A paired-sample t-test revealed that there were no significant differences as a function of demonstrator description or as a function of the outcome (Shock or No shock). The regions indicated during observation of the demonstrator’s choice and/or the outcome of that choice are typically associated with social processes and learning and decision-making more generally. For example, the middle temporal cortex has been shown to be involved in action observation and imitation^29^ and the medial superior frontal cortex, as well as the inferior superior frontal cortex, have been implied in both social and non-social decision-making and learning^10^. Our results indicate that the behavioral effect of demonstrator description was likely not driven by differences in the perception of the demonstrators’ actions, for instance increased attention directed towards the decisions made by the Best demonstrators, or the outcome of those decisions.

For the action phase, during the presentation of the choice stimuli, participants showed increased brain activity in the left middle temporal cortex when the demonstrator was described as Worst (but not Best) as compared to baseline (see Table 1). The left middle temporal cortex has been implicated in cognitive mentalizing^30^. However, activity in this region did not differ as a function of the demonstrator’s described ability.

Our behavioral as well as RL modeling analyses indicated that demonstrator description affected copying. For the model-based analysis, we therefore analyzed the effects of the copying rate (i.e., model parameter *κ*). Our analysis showed that the copying rate significantly modulated participants’ brain activity during the presentation of the choice stimuli when the demonstrators were described as Best, but not Worst. These effects were seen in the bilateral supramarginal gyrus, left middle temporal cortex, and right inferior temporal cortex, regions implicated in action observation and imitation^29^. We also noted effects in the right middle cingulum, a region involved in both social and non-social decision-making and conflict monitoring^10,31^.

We did not find any modulatory effects of either the observational or the individual prediction error during the observational or action phase, respectively.

## Discussion

In a previous behavioral study, using a similar task, but where both the described and the actual ability of the demonstrator was varied, we demonstrated that performance was higher following observation of a demonstrator described as having a high ability. Those results suggested that this effect was driven by differences in what we here refer to as observational outcome learning, rather than differences in copying. Here, we aimed to 1) conceptually replicate these behavioral effects of described demonstrator ability on observational learning, and 2) investigate the neural correlates underlying this biased observational learning by examining regions linked to social cognition and attention, in particular mentalizing and observational learning (e.g., the dlPFC, dmPFC, vmPFC and the temporoparietal regions). We hypothesized that describing the demonstrator as having high ability (Best), compared to low (Worst) would lead to higher performance, an effect we expected to be driven by differences in observational outcome learning rather than copying. On a neural level, we wanted to investigate such biased learning by testing if activity in the regions of interest differed with the described ability and/or relevant latent variables derived from a RL model of participants’ behavior.

Interestingly, we saw no tendency for performance to be higher following observation of demonstrators described as having high compared to low ability and we did thus not see the same behavioral effects as in our earlier study^12^. Instead, post-hoc analyses showed that copying was sensitive to the description of the demonstrators’ ability, such that participants copied demonstrators described as high in ability more than those described as low in ability. Results from our RL models add further details to these findings. First, our analyses show that nearly all participants learned from observation and that the difference in copying as a function of demonstrator description included negative copying, avoidance, of choices made by demonstrators described as low in ability. Secondly, by dividing participants into two main subgroups of participants, we could demonstrate that the copying effect appears driven by one of these subgroups, whereas participants in the other subgroup did not copy. We do not know if these subgroups of individuals differ in other aspects. Future experiments could investigate such differences in characteristics or traits. One could for instance imagine that individuals that have a lower belief in their own ability, or that have poorer working memory, have a tendency to apply a copying strategy, since such a strategy might (appear to) be less cognitively demanding and in some sense would provide a learning short-cut. A comparison between the matching conditions in the present and our previous^12^ study, showed that when the demonstrator was described as having low ability, performance in the two experiments did not differ. When the demonstrator was described as having high ability, however, performance was superior in our previous study. This difference between the two experiments seems to be driven by the subgroup of participants in the present study that also copy (see Supplementary Information). Although we do not know what causes these differences between participants, the findings appear to be in line with research suggesting that individuals differ in their use of social learning strategies, where some appear to focus only on the behavior of their peer’s while others also incorporate information about their payoffs^32^.

The reasons for the different observations between the present study and our previous work are not entirely clear. Variations in the experimental setup may provide some insight, as the paradigms, while similar, are not identical. The most obvious difference is that the present study took place in an MR scanner, an environment that can be stressful to participants, possibly resulting in higher levels of anxiety and release of cortisol^33^. Although unknown if this is the case here, it is possible that increased anxiety might have made participants rely more heavily on only the choices of the demonstrators for learning. This would have led to copying, rather than observational outcome learning, as this might be more cognitively demanding. Another difference between the studies is that in the present, the probabilities to receive a shock as a function of choice varied between blocks, while in the previous they did not. On average, the difference between the probabilities in a block was smaller in the present study compared to the previous. Taken together, these differences made the task in the present study more difficult, something that might have made some participants rely on copying to a larger extent in the present study, in line with “copy when uncertain” biases as suggested by Rendell^4^. The fact that described ability varied within participant in the present study, rather than between as in the previous, is another factor that might have enhanced this effect further. Interestingly, possible effects of the differences in the experimental setup might also explain the differences in behavior between the two identified subgroups in the present study, by influencing the behavior of some, but not all, participants.

Even though our present findings were not all aligned with our earlier results, we demonstrated description-sensitive observational learning, motivating us to investigate the neural underpinnings of this effect of described demonstrator ability. The analyses of the neural correlates underlying the description-sensitive observational learning evident in our behavioral data did not reveal any clear effects of description of demonstrator ability. During the observation phase, we saw increased activity in brain regions involved in action observation and imitation, as well as decision-making and learning (both social and non-social). Demonstrator description did, however, not modulate these brain responses. Interestingly, during the presentation of the choice stimuli in the action phase, we noted an increased activity in regions related to mentalizing when demonstrator ability was described as high, but not low. Our analyses further demonstrated that brain activity in regions linked to action observation and imitation, as well as decision-making, was modulated by the copying rate during presentation of the choice stimuli when the demonstrator was described as high, but not low, in ability. One possible explanation for the null results with regards to the effects of demonstrator description could be that the behavioral differences between the two conditions were driven by more subtle neural dynamics than our current fMRI analyses could capture. Due to time constraints, our paradigm was not optimized to capture neural signals of individual prediction errors, which is likely the main reason for why we did not observe such activity. The absence of prediction errors in our fMRI data may also be related to the specific model used. This model, which was not a clear winner, may not have captured the neural processes closely enough. Even though additional research is required to verify our results, our findings suggest that mentalizing processes combined with more general learning and decision-making processes drive the effects of biased observational learning. Our results do not lend support to the hypothesis that differences in attention to the decisions made by the demonstrators play a key role in the behavioral effects.

In our fMRI analyses, the conclusions about psychological processes that we draw from brain activity are based on prior literature. This method has its weaknesses and could be considered inferential in nature. Our results should therefore be interpreted with care. For findings to be more precise, future studies could, for example, use functional localizers to identify ROIs related to social cognition and/or mentalizing processes.

It is unclear to what extent our findings would generalize to a real-life context. In real-life contexts, consequences of decisions are often delayed or obscured from an observer’s point of view, likely increasing reliance on description of others’ abilities or reputation. It is further worth noting that, given that we failed to find support for our hypothesis based on previous findings, several of our results are based on post-hoc analyses, and should be interpreted with caution. A replication of these findings is needed to establish reliability in our results. Nevertheless, our results clearly show that descriptions of others ability not only influence how their performance is perceived, but also how we learn from them. Such description-sensitive observational learning can prove to be adaptive and efficient, but it might also lead us down the wrong path, for instance when we copy behavior of others that perform suboptimally.

## Methods

### Participants

Forty-five participants were recruited to the experiment approved by the local ethics committee (Regional Ethics Board in Stockholm, 2012/340-31/4). The sample largely consisted of university students. A priori power calculations based on a behavioral pilot study indicated that 35 participants were needed to reach statistical power of 80%. Inclusion criteria were: right-handedness, normal or corrected to normal vision using contact lenses and absence of psychiatric or psychological disorders. Three participants canceled their participation during data collection and data from four participants were excluded due to technical problems, leaving a final sample of thirty-eight participants (19 males, mean age = 26.5 years, sd = 5.08). Of these, one additional participant was excluded from the fMRI analyses due to incomplete neuroimaging data. Thus, thirty-seven participants were included in the fMRI analyses (18 men, mean age = 26.49 years, sd = 5.10). All participants gave written informed consent and were paid for their participation.

### Material

**Fig. 5:**
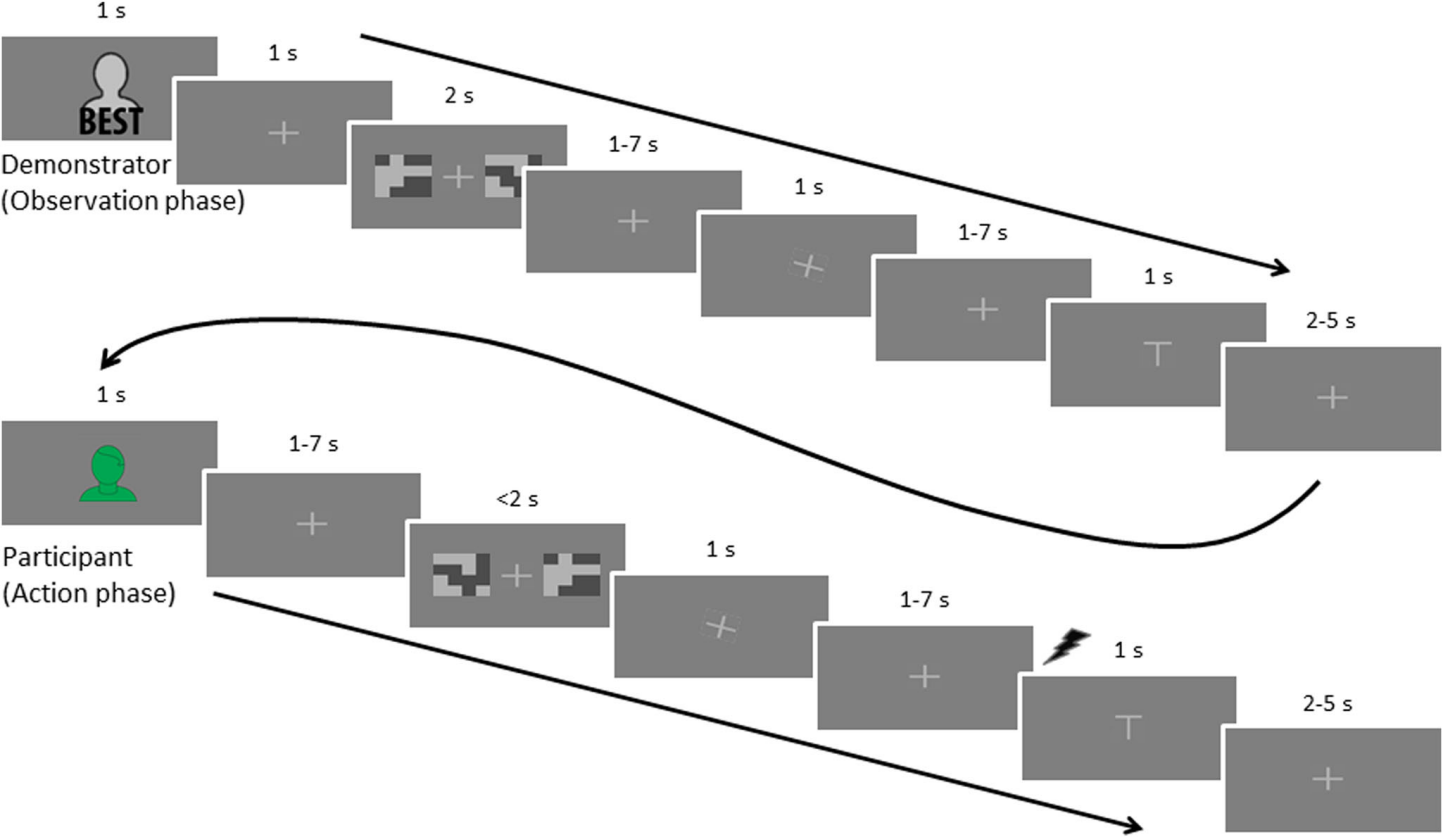
Trial setup. Each phase began with a presentation of a figure indicating whose turn it was. This was followed by a fixation cross and then the two pictures indicating the choice options were shown. In the observation phase, first the pictures for the choice options were shown, followed by a fixation cross of jittered duration before the demonstrator’s choice was shown, indicated by the fixation cross tilting 20° towards the left or right (to indicate left or right choice). Jittered durations were randomly sampled from a Poisson distribution, with removal of durations outside of the wanted range. Jittered durations ranging between 1 and 7 s were drawn from a distribution with a mean of 2, and durations ranging between 2 and 5 s were drawn from a distribution with a mean of 2.5. In the action phase, the pictures were shown for a maximum of 2 s, and the presentation terminated when the participant made their choice. The participant’s choice was indicated as for the demonstrator, with the cross tilting 20° towards the left or right, but without being preceded by a neutral fixation cross. In both phases, the fixation cross was then shown in neutral position before the outcome of the choice was indicated on the screen with a T-shaped symbol for the shock and the same symbol turned upside down when there was no shock. During the action phase, an electric shock was delivered to the participant at the onset of the presentation of the symbol for shock. No shock was administered during the observation phase or during training.

Participants performed a simple probabilistic two-choice task to avoid shock similar to the task used in the previous study. Choice options were indicated visually using neutral abstract pictures of equal luminescence, see Fig. 5. In total, there were 40 different pictures, out of which each participant observed a random subset of 18. The computerized task was programmed in Python v 3.6 with the help of PsychoPy v 1.83. Shocks consisted of 100 ms DC-pulse electric stimulation (administered using the STM200; BIOPAC Systems) applied to the participant’s left wrist. Choices were made by the right hand using a button pad. Eye-tracking (iView, SensoMotoric Instruments) was used to collect pupil data. Due to technical problems, eye-tracking data for eighteen of the thirty-eight participants were either missing or of very poor quality and data for the remaining twenty participants were in many cases of relatively poor quality. Therefore, no further analysis was performed on the eye-tracking data.

### Instruction and preparation

Before being placed in the scanner, participants were instructed that during the task, they should choose one of the two pictures that were repeatedly presented on the screen. One of the two would be associated with a higher probability than the other of being followed by an uncomfortable shock. Participants were told that their task was to try to avoid getting a shock and that they would observe the choices of previous participants (the demonstrators), who had carried out the same task earlier, although without the possibility of observing anyone else. The demonstrators were described to vary in their ability to learn the task, and based on their ability, they had been categorized into two equally sized groups described as having greater (Best group) or worse (Worst group) ability to learn the task. We further told the participants that for each new pair of pictures to choose between, we would randomly draw a previous participant from one of the two groups whose choices and outcomes the participant could observe. However, unbeknownst to the participant, the choices that the demonstrators made were always random and thus, observed performance was always low. In order for the participants to keep track of when it was their turn and when it was the demonstrator’s turn, participants selected an avatar (out of nine available) to represent themselves during the task. See Supplementary Information for instructions and depictions of the avatars.

Before entering the scanner, a workup procedure was carried out to calibrate the strength of the shocks so that they were uncomfortable but not painful. Participants spent ~1 h in the scanner. First, we carried out calibration of the eye-tracker, and ran initial scans. Participants then read instructions and carried out a practice session. After approximately 15 min, they started with the experimental task, which took around 35 min to complete.

### Experimental procedure

First, to familiarize themselves with the task, participants carried out a training block of six trials where they observed a “neutral” demonstrator, allegedly drawn randomly from the entire pool of previous participants (but which made random choices). Next, participants proceeded to the proper experiment, consisting of eight blocks, each containing eight trials, see Fig. 5. To vary described demonstrator ability within participants, demonstrators were described as Best during half of the blocks, and as Worst for the other half of the blocks. The order of these blocks was randomized such that for each subsequent pair of blocks one allegedly showed a demonstrator from the Best group and one from the Worst.

For each block, the participants had to repeatedly select one of two choices in order to try to avoid shocks. Each choice was associated with a probability of being followed by a shock and within each pair of choices these probabilities differed such that one was the optimal choice. To promote the need to observe outcomes from both choices to identify the optimal choice, the probabilities of getting a shock following a choice were randomized so that they varied between blocks. This also resulted in blocks with varying difficulty since the difference in probability of receiving a shock from each of the two available choice options differed between blocks. A smaller difference likely made it more difficult to learn which choice option was the best to select while a larger difference would have made it easier. To balance the task between participants and conditions, we kept the overall threat level (probability of receiving a shock) and difficulty level the same for each participant and demonstrator description. This was done by constraining the probabilities so that the mean overall probability of getting a shock throughout the experiment, given random behavior, was 0.5 for each participant and demonstrator description, and so that the mean difference between the higher and the lower probability (which guides the difficulty of the task) was 0.5 for all participants and demonstrator descriptions. In addition, the difference between the higher and lower probability of getting a shock was restricted within the range of [0.3, 0.7] so that learning would not be too easy or too difficult in any block. For instance, a block where the probabilities of receiving a shock was 0.25 and 0.62, respectively, would be considered a harder block, as the difference (0.37) is small. A block where the probabilities of receiving a shock instead was 0.09 and 0.73, would be considered an easier block, as the difference (0.64) is larger. Given these restrictions, the probabilities of getting a shock in our collected data sample ended up within the range of [0, 0.65] for the optimal choice and [0.39, 1] for the suboptimal choice. For the training block, the probabilities of getting a shock were set to 0.75 and 0.25 respectively, so that the mean probability of shock was 0.5 and the difference in probability was 0.5. For further details regarding the variability in the probability of receiving a shock, see Supplementary Information.

To promote engagement in the task, in case the participant failed to indicate his/her choice within a 2-s time window, no choice was indicated and the probability of receiving a shock was set to 0.5 (participants were instructed that the probability of a shock would be high if they failed to indicate a choice, without further details). Shocks were administered to the participant following the participant’s own choice, not during observation of the demonstrator, with the exception of the training block where no shocks were administered in either case. Shocks and absence of shocks were also indicated visually: a T-shaped symbol indicated shock and the same symbol turned upside down indicated no shock. This way, participants could see when the demonstrator received a shock or not while also keeping the visual setup constant regardless of whether it was the demonstrator or participant who made a choice (important for collection of the later discarded eye-tracking data). Each trial consisted of an initial observation phase, during which the demonstrator made a choice and allegedly received an outcome (shock or no shock), followed by the action phase, during which the participant him-/herself made a choice and received an outcome, see Fig. 5.

After finishing the experiment, participants filled out a set of questionnaires to assess how they estimated the average performance of the demonstrators described as Best and Worst, as well as their own performance, all on a scale from one (“Very poor”) to five (“Very good”). They also rated how big advantage on a scale from one (“No advantage”) to five (“Big advantage”) they felt that it had been to observe the demonstrators. Finally, we used a funneled debriefing procedure to get a verbal report on the strategy the participants used to learn and make choices (see Supplementary Information) and whether or not they believed that the demonstrators were indeed previous participants, before informing them that the previous participants had not been real and that the behavior of the demonstrators had been programmed in advance.

### Behavioral analyses

All statistical analyses of behavioral data were conducted using R Statistical Software, v 4.1.1^34^. Performance ratings were analyzed to test whether demonstrator description influenced perceived demonstrator ability. Performance ratings consisted of ordinal data, and therefore analyses of demonstrator performance ratings were carried out by fitting Cumulative Link Models using the ordinal package^35^. The logit link function and a flexible threshold structure were used. Since actual performance was also likely to have influenced ratings, we further ran two separate analyes that, in addition to demonstrator description, also included one of two measures of the observed demonstrators’ mean performance as predictors, one direct and one indirect. The direct measure of performance was the proportion of optimal choices made by the demonstrators throughout the task. The indirect measure of performance was the proportion of shocks received by the demonstrators throughout the task. Both predictors were centered on the theoretical means, which was 0.5 for both. One participant lacked data on ratings and was thus excluded, leaving a sample of 37 participants to be included in the analyses. Analyses of the participants’ choices were carried out by fitting Generalized Linear Mixed Models (GLMMs) using the *lme4* package^36^. Here, the dependent variable was participant choice, coded according to optimality, based on which choice option was assigned the lowest probability of being followed by a shock (Optimal choice = 1, Suboptimal choice = 0). Cases when the participant failed to make a choice were excluded from the analyses. Across participants, the number of missed choices was at most six per participant, with a median of one missed choice and with most participants having no missed choices. For the GLMMs, the binomial distribution with logit link function was used. All GLMMs included by-subject random intercepts and random slopes for all predictors. The estimation method was Laplace Approximation and the covariance structure was unstructured. For all statistical tests on behavioral data an alpha level of 0.05 was used. All reported *p*-values are two-tailed. Model factors were tested using the type II Wald Chi-square test.

### Reinforcement Learning modeling

We fitted a set of RL models using R^34^ to participants’ behavior in order to get a more mechanistic and fine-grained understanding of participants’ behavior and to be able to extract latent variables of learning to be used in the fMRI analyses. The RL models were based on the Q-learning algorithm^37^ and modified to include both copying and observational outcome learning^9,12^. According to the Q-learning algorithm, expected values of the two available choices are represented by *Q*-values, ${Q}_{i=\mathrm{1,2}}$. These *Q*-values are updated following the outcome of own choices and guide subsequent decisions. *Q*-values were initialized as 0 for both choices. Outcome was coded as either −1 (shock) or 1 (no shock). To include observational outcome learning, updating of the *Q*-values also follows after the observation of the demonstrator’s choice and the subsequent outcome, as described below. In the course of one trial, the *Q*-values are thus updated twice, first following the observational phase, after half the trial has passed, and then following the action phase, at the end of the trial. At the observational phase of a trial *t*, an observational prediction error, *PE*_*observation*_, is used to update the *Q*-value at trial *t* + 0.5 for the observed demonstrator choice (*D.Choice*) using the standard delta rule with an observational learning rate *α*_*observation*_. The *Q*-value for the unselected option remains unchanged:

1 $$P{E}_{{observation}}\left(t\right)={Outcom}{e}_{D.{Choice}}\left(t\right)-{Q}_{D.{Choice}}\left(t\right)$$

2 $${Q}_{D.{Choice}}\left(t+0.5\right)={Q}_{D.{Choice}}\left(t\right)+{\alpha }_{{observation}}* P{E}_{{observation}}\left(t\right)$$

3 $${Q}_{{{\neg }}D.{Choice}}\left(t+0.5\right)={Q}_{{{\neg }}D.{Choice}}\left(t\right)$$

Following the observation, the model now calculated the probabilities that the participant will select each choice. This is done in two steps. First, the model uses the softmax decision function with the temperature parameter *β* to calculate the probabilities that the participant will select each choice given the *Q*-values (after half a trial, at *t* + 0.5):

4 $${p}_{i}\left(t+0.5\right)=\frac{exp \left({Q}_{i}\left(t+0.5\right)/\beta \right)}{{\sum }_{j=1}^{2}exp \left({Q}_{j}\left(t+0.5\right)/\beta \right)}$$

Next, the probabilities are updated by including copying. Copying is modeled as a tendency to make the same choice as the demonstrator, using a copying parameter *κ* (note that if *κ* *=* 0, no copying occurs and the probabilities are left unchanged):

5 $${p}_{D.{Choice}}\left(t+1\right)={p}_{D.{Choice}}\left(t+0.5\right)+\kappa * \left(1-{p}_{D.{Choice}\left(t+0.5\right)}\right)$$

If *κ* *<* 0 this might result in negative probabilities. If this occurs, the probability is set to 0.

6 $${p}_{{{\neg }}D.{Choice}}\left(t+1\right)={1-p}_{D.{Choice}}\left(t+1\right)$$

Next, participants learn from the outcome of their own choice similarly as during the observation phase. An individual prediction error, *PE*_*Individual*_, is used to again update the *Q*-values of the participant’s own choice (*P.Choice*), using an individual learning rate, *α*_*Individual*_.

7 $$P{E}_{{Individual}}\left(t\right)={Outcom}{e}_{P.{Choice}}\left(t\right)-{Q}_{P.{Choice}}\left(t+0.5\right)$$

8 $${Q}_{P.{Choice}}\left(t+1\right)={Q}_{P.{Choice}}\left(t+0.5\right)+{\alpha }_{{Individual}}* P{E}_{{Individual}}\left(t\right)$$

9 $${Q}_{{{\neg }}P.{Choice}}\left(t+1\right)={Q}_{{{\neg }}P.{Choice}}\left(t+0.5\right)$$

To systematically vary inclusion of observational outcome learning and copying as well as description-sensitive learning, a set of 50 RL models were generated by varying how the social learning parameters, *α*_*observational*_ and *κ*, were modeled (included/not included, description-sensitive/description-insensitive, for details, see Supplementary Information). Depending on parameter setup, the models could thus be labeled as either non-social learning models (1 model), pure observational outcome learning models (9 models), pure copying models (4 models), or mixed strategy learning models (36 models). Models were also labeled as description-sensitive or description-insensitive depending on whether or not observational learning was sensitive to the described ability of the demonstrator, essentially by allowing the observational learning parameters of interest, *α*_*observational*_ and *κ*, to differ as a function of described ability. All models included individual learning, thus all models included *α*_*Individual*_ as well as *β* as free parameters.

All models were fit individually to each participant’s choices over all trials by minimizing the negative log-likelihood, -*neg*(*L*). Model fitting was carried out using the *mle2* function from the *bbmle* package, with the *optim* optimization function and the *BFGS* optimization method^38^. The learning rates (*α*_*Observation*_, *α*_*Individual*_) were constrained within the interval [0, 1] while *β* was constrained within [0, 5]. The copying rate, *κ* was constrained within [−1, 1], allowing also for negative copying, or avoidance, a decreased probability of selecting the same choice option as the demonstrator. Model comparisons were based on the Akaike Information Criterion, AIC, a measure of the goodness of fit of a model that also takes the complexity of the model into account^39^. AIC is calculated from the number of free parameters, *θ*, in the model and the log-likelihood, *log(L)*:

10 $${AIC}=2\theta -2{log} (L)$$

Mean AIC over participants was used to identify the best model of a set of models, with the lowest AIC being the best.

### fMRI acquisition and preprocessing

Participants were scanned with a 3-T GE MR750 scanner using an 8-channel head coil and a gradient echo pulse sequence (repetition time = 2.40 s, echo time = 28 ms, flip angle = 80°, field of view = 288 mm). Forty-seven slices (slice thickness = 3 mm) were collected in axial orientation covering the whole brain. Before image acquisition, 5 dummy scans were performed to allow for MR stabilization. In total, 242 volumes were collected for each of the four functional runs. Before fMRI, a T1-weighted MRI scan was collected from each participant using the Ax 3D BRAVO sequence (repetition time = 7.9 ms, echo time = 3.1 ms, field of view = 240 mm, 176 adjacent sagittal slices, slice thickness = 1 mm).

The data were preprocessed and analyzed in SPM12 (Functional Imaging Laboratory, Wellcome Department of Imaging Science, www.fil.ion.ucl.ac.uk/spm/) implemented in MATLAB R2019b (The MathWorks Inc., Natick, MA). After slice timing correction, images were realigned to the volume acquired immediately before the anatomical scan, using six parameter rigid-body transformations. They were then coregistered with the structural data, normalized to standard space using the Montreal Neurological Institute (MNI) template with a voxel size of 2 × 2 × 2 mm, and smoothed using a Gaussian kernel with an isotropic full-width-half-maximum of 6 mm.

### First-level analysis

Statistical parametric maps were generated for each individual within the framework of the general linear model (GLM) in SPM12. Event onsets were modeled with a stick function and convolved with the hemodynamic response function. Events were specified as occurring at the start of stimulus presentation or at a button press, respectively. Trials that participants did not respond to were excluded from the analysis. We concatenated the runs by treating all four as a single time series, because for some participants not all runs included all events of interest, and could therefore not be modeled. We set up two different models, one to investigate the effects of Demonstrator description, and another to investigate signals of prediction errors derived from RL modeling.

For the analyses focusing on the effect of Demonstrator description, 23 events of interest were modeled, corresponding to both the observational phase at the beginning of each trial and the action phase at the end. More precisely, for the observational phase, events (presentation of the avatar, presentation of the choice stimuli, and presentation of the choice and outcome respectively) were modeled as functions of Demonstrator description (Best or Worst), Choice (Optimal or Suboptimal) and Outcome (Shock or No Shock), resulting in 12 regressors. For the action phase, we model the corresponding setup of events and all of these, apart from the presentation of the avatar, were similarly modeled as a function of Demonstrator description (Best or Worst), Choice (Optimal or Suboptimal) and Outcome (Shock or No shock). In addition, in a post-hoc analysis, we wanted to explore the effects of model-derived copying. To do this we parametrically modulated the presentation of the choice stimuli by the copying parameter *κ* from the full (maximum) RL model (see Supplementary Information for details). We reasoned that this was the time point where participants most likely contemplated about their choice and that copying tendencies might influence brain activity here. For the action phase, we ended up with 11 regressors and two parametric modulators. Six motion parameters estimated during realignment and two dummy regressors to control for effects between runs were also included in the model as regressors of no interest.

For the analyses of the prediction-error signals, we modeled 19 events of interest, corresponding to both the observational phase and the action phase. For the observational phase, the presentation of the avatar, presentation of the choice stimuli, and presentation of the choice were modeled as a function of Demonstrator description (Best or Worst). The presentation of the outcome was modeled as a function of Outcome (Shock or No shock) and was further parametrically modulated by participants’ observational prediction errors. This resulted in eight regressors for the observational phase. Events of the action phase (presentation of the choice stimuli and the button press indicating choice) were modeled as a function of Demonstrator description (Best or Worst) and the presentation of the outcome was modeled as a function of Outcome (Shock or No shock). The presentation of the outcome was further parametrically modulated by the participants’ prediction errors, resulting in nine regressors for the observational phase. Six motion parameters estimated during realignment and two dummy regressors to control for effects between runs were also included in the model as regressors of no interest.

### Second-level analysis

Analyses were performed within the framework of the general linear model. SPMs were generated using t-statistics and a threshold of *p* < 0.001. Results were considered significant at a family-wise error-corrected cluster-extent threshold of *p* < 0.05.

**Fig. 6:**
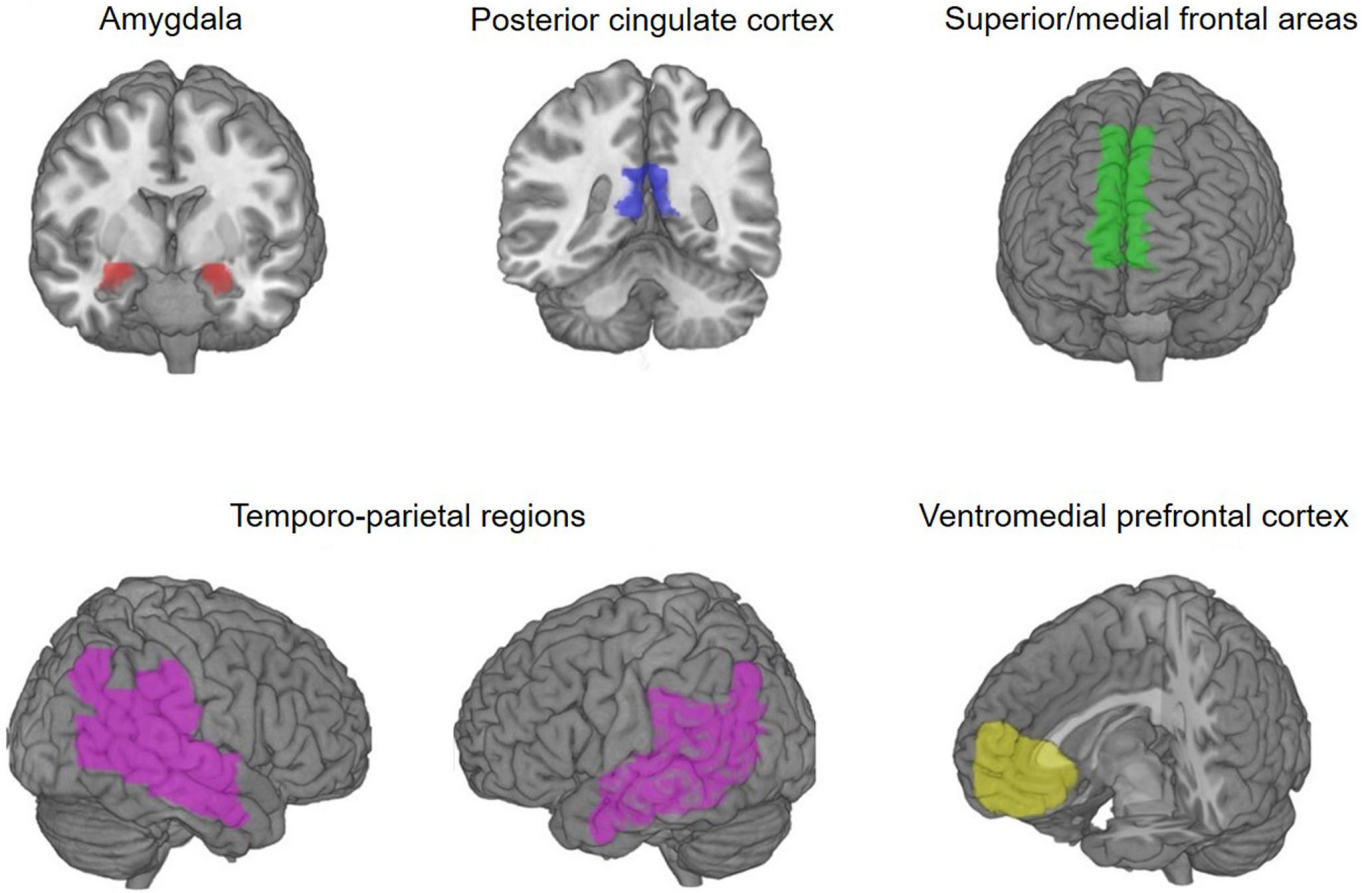
Regions of interest. Illustration of the regions of interest that were combined into one binary mask and used as an explicit mask when analyzing effects of Demonstrator description.

Given their involvement in observational learning and theory of mind^9,26^, we specified as regions of interest the dorsolateral and dorsomedial prefrontal cortex (dlPFC and dmPFC), the ventromedial prefrontal cortex (vmPFC), the tempo-parietal region (specifically the tempo-parietal junction and superior temporal sulcus), the posterior cingulate cortex (PCC), and the amygdala. The masks for the dmPFC, PCC, amygdala, and temporoparietal region, which included the superior and middle temporal gyrus, the angular gyrus and supramarginal gyrus were created using the automated anatomic labeling atlas (AAL; Tzourio-Mazoyer et al.^40^). The dlPFC mask was created by adding a 20 mm sphere around a peak coordinate reported in Burke et al.^9^ and multiplying this sphere with a PFC mask created with the AAL to make sure it only covered regions within the brain (i.e., MNI coordinate 48 30 27)^9^. In addition, we used an anatomical mask of the vmPFC from the Anatomical Masks collection (https://neurovault.org/collections/1290/) on NeuroVault (https://identifiers.org/neurovault.image:18650). All masks were combined to a bilateral binary gray-matter mask that was used as an explicit mask in all group-level analyses (see Fig. 6).

To investigate the effect of Demonstrator description on brain activity we performed a set of paired sample t-tests in SPM. For the observation phase, we first compared brain activity during the presentation of demonstrator’s choice when the demonstrator was described as being from the Worst group (compared to baseline) versus the Best group (compared to baseline). This was done to investigate whether participants processed the demonstrator’s choices differently depending on the demonstrator’s description. Second, we examined brain activity during the presentation of the outcome of the demonstrator’s choice when the demonstrator was described as being from the Worst group (compared to baseline) versus the Best group (compared to baseline). For the action phase, we first compared brain activity during the presentation of the stimuli when the demonstrator was described as being from the Worst group (compared to baseline) versus the Best group (compared to baseline). This was done to investigate whether the processing of the stimuli depended on the demonstrator’s description. We further performed Best > Worst and Worst > Best contrasts for each phase (i.e., for demonstrator’s choice, demonstrator’s outcome, and stimulus presentation). Finally, we investigated how brain activity during the presentation of the choice stimuli was modulated by the participants’ copying behavior (RL model parameter *κ*) when the demonstrator was described as being from the Worst group and further compared it to a potential modulation when the demonstrator was described as being from the Best group.

Based on previous findings^41^, we used the striatum as region of interest for the analyses of prediction errors. The striatum mask was created by combining separate caudate, putamen, and accumbens masks from the AAL atlas^40^, using the Marsbar toolbox (https://marsbar-toolbox.github.io).

To investigate the existence of prediction-error signals in brain activity, we performed two sets of paired sample t-tests in SPM. For the observational phase, we investigated how brain activity during the presentation of the outcome was modulated by the participants’ observational prediction error. For the action phase, we investigated how brain activity during the presentation of the outcome was modulated by the participants’ prediction error.

## Supplementary information

Supplementary Information

## Data Availability

The behavioral data (choice data) and participant data for this study have been made publicly available at the Open Science Framework (OSF) and can be accessed at (https://osf.io/6xe8v/?view_only=58861b1f80af4594a723459c91ef89b7). The neuroimaging data is available upon reasonable request from the corresponding author.

## Code availability

The code for data collection and analyses may be made available to qualified researchers upon reasonable request.
