# Supplementary material for: Effects of described demonstrator ability on brain and behavior when learning from others
Source: NPJ Sci Learn. 2025 Jan 16;10:4. doi: 10.1038/s41539-024-00292-0 (PMC11739481; doi:10.1038/s41539-024-00292-0)
Supplement: Supplementary file 1 — Supplementary Information [file 41539_2024_292_MOESM1_ESM.pdf]

Supplementary Information

Effects of Described Demonstrator Ability on Brain and Behavior when Learning from Others

Ida Selbing[1], Nina Becker[1], Yafeng Pan[1,2], Björn Lindström[1], Andreas Olsson[1]

[1] Division of Psychology, Karolinska Institutet, Solna, Sweden

[2] Department of Psychology and Behavioral Sciences, Zhejiang University, Hangzhou, China

Instructions

Participants were given the following instructions.

The general task:

- You are going to complete a task where you are presented with pairs of pictures several times. You will also observe the choices that previous participants made in the same situation.
- When you are presented with a pair, choose one of the pictures. Following your choice there is a probability that you will get an electric shock. The probability is determined by what picture you choose. One of the pictures will result in a higher probability of receiving a shock and the other will result in a lower probability.
- Your task is to avoid receiving shocks by choosing the picture with the lowest risk of resulting in a shock.

Failing to select at choice option:

- If you fail to make a choice, the cross in the middle will remain unchanged
  ...and you run a high risk of receiving a shock.

The demonstrators:

- We let a number of participants try this task earlier. They varied quite a lot in how well they learned to avoid shocks.
- We are interested in how you can learn this task if you have access to the choices of another person. Therefore, you will watch how a previous participant made his or her choice in the same situation before you get to choose yourself.
- Based on their abilities to learn to avoid shocks, previous participants were split into two equally sized groups – those who learned well (the Best group), and those who learned poorly (the Worst group).
- For each pair of pictures you will choose between we are going to randomly draw a participant from either the Best or the Worst group, and you will see that participant's choices.

Avatars

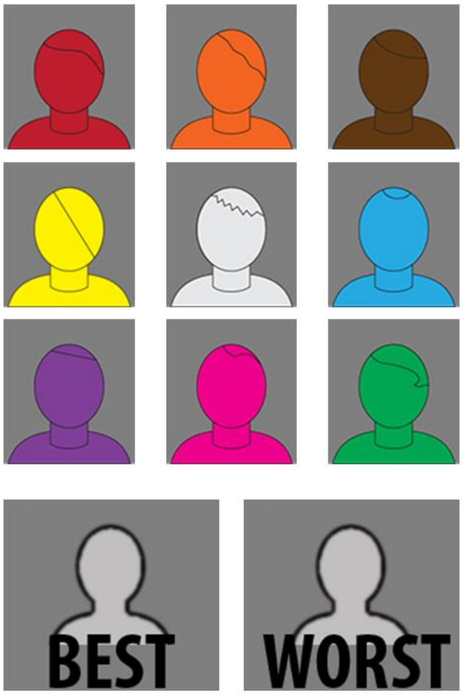

Supplementary Figure 1. Avatars.

Participants selected which picture should represent themselves out of the top nine colored avatars. The bottom row demonstrates how the demonstrators were depicted.

Experimental paradigm – details

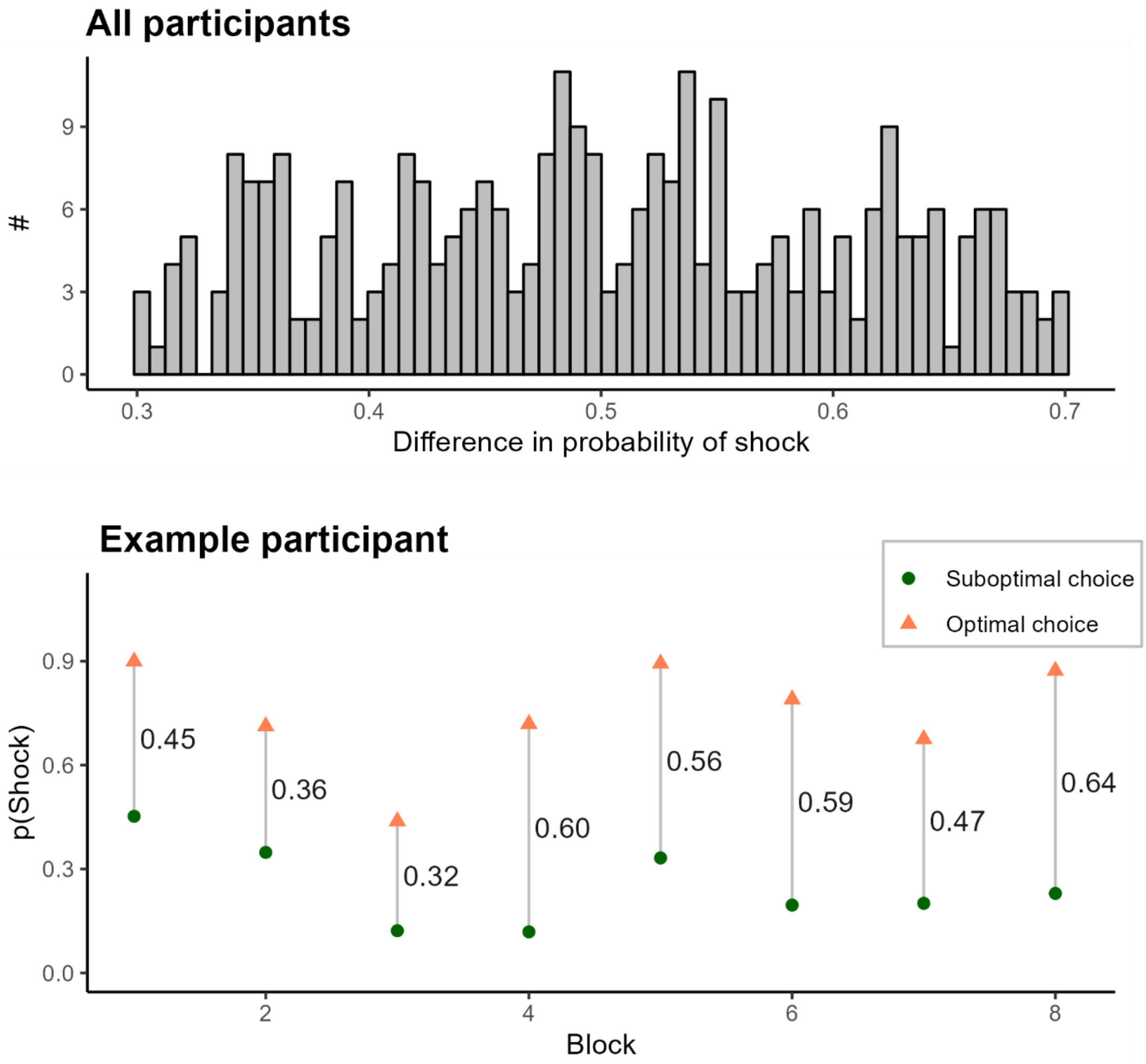

Supplementary Figure 2. Differences in probability of shock.
The top panel shows the distribution of the difference in probability of shock given a suboptimal versus optimal choice across all participants and blocks. The bottom panel shows the probability of shock following a suboptimal or optimal choice for each block of one single participant. The difference between these two probabilities is demonstrated by a line with an accompanying difference measure.

Supplementary Table 1. Study differences.

Summary of the differences between the present study and the previous study[1] upon which the present study is based.

Previous study	*Present study*
No scanner	*Scanner*
Varied actual (within) and described (between) ability	*Varied described ability (within), actual ability always low*
80 trials total	*64 trials total*
Timing:<br><br>*In general trials were shorter (approx. 22 s)*<br><br>*e.g. Demonstrator choice shown 0.5 s after end of stimuli presentation*	*Timing:*<br><br>*In general trials were longer (approx. 30 s)*<br><br>*e.g. Demonstrator choice shown 1 - 7 s after end of stimuli presentation*
Non-aversive sound stimuli (no visual stimuli) to indicate demonstrator shock	*Visual symbol to indicate demonstrator shock (same as when shock to oneself)*
Questions between blocks – "How many shocks did the demonstrator receive during the previous block?"	*No questions between blocks (in an attempt to decrease trial duration + in order to enhance effect of description by making participants less aware of demonstrator's actual ability)*
Fixed probability of shock for Suboptimal/Optimal choice (0.2/0.8)	*Probability of shock for Suboptimal/Optimal choice varied between blocks*
The difference in shock probability, i.e. p(shock|suboptimal chocie) - p(shock|optimal choice), was always 0.6.	*The mean difference in shock probability, i.e. p(shock|suboptimal chocie) - p(shock|optimal choice), over all blocks a participant carried out was always 0.5 (more difficult).*

RL models – parameter recovery

For the three models that were of greatest interest for our analyses, we carried out parameter recovery[2] to investigate how well we could recover fitted parameters from a simulated dataset where parameters were known.
To do this, we first simulated behavior of 200 "virtual participants". The probabilities of getting a shock following the optimal/suboptimal option were taken from the actual experimental data but the demonstrators' choices and the outcomes of those choices were simulated. Since it is recommended to try to recover parameters within the same range as those fitted[2], parameters were drawn from a uniform distribution that was restricted to be within the same range as 80% of the fitted parameters for each model (highest 10% and lowest 10% were removed). Parameter recovery was carried out using the exact same method as was used for model fitting (see the Reinforcement Learning modeling section in main text). The Pearson correlation coefficient was used as a measure of the correlation between the simulated and recovered parameter. See Supplementary Figure 3 for an overview of simulated and recovered parameters of the three models of interest.

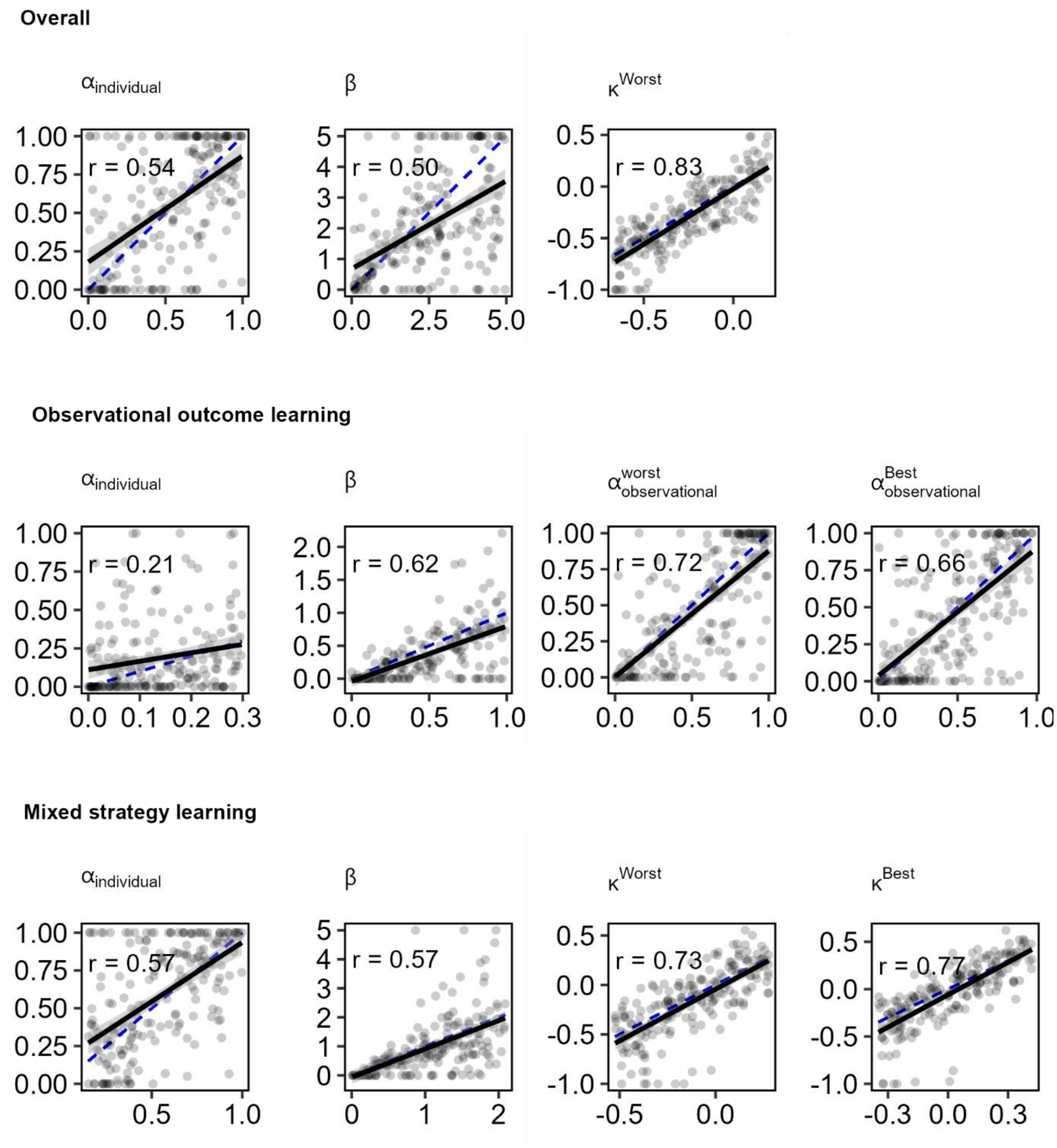

Supplementary Figure 3. Parameter recovery.

Results from the parameter recovery of the three models of interest. Simulated parameters are plotted on the x-axes and recovered parameters on the y-axes. The black solid line indicates the linear relationship between the simulated and recovered parameters. The blue dashed line indicates perfect correlation (r = 1). Note that the scale of the axes varies between as well as within plots.

RL models – details

As previously described, we ran a large set of RL models which allowed us to systematically vary inclusion of the two forms of learning (copying and observational outcome learning) as well as description-sensitive learning. Copying was thus modelled using two parameters, κ^{Best} and κ^{Worst}, these could be either set to 0 or modelled as one or two free parameters. This allowed for 4 combinations or ways to include copying in our models. Observational outcome learning was also modelled using two parameters, $\alpha^{Best}_{Observational}$ and $\alpha^{Worst}_{Observational}$ and these could in a similar way be either set to 0 or modelled as free parameters. We also assumed that learning from observed outcome could be either similar or different from learning from own outcome. This allowed for 9 combinations or ways to include observational outcome learning in our models. Altogether, this allowed us to test 50 RL models, see Supplementary Table 2.

Supplementary Table 2. Overview of the different models and their performances.
X means the parameter is included as a free parameter in the model. The observational learning rate can be set to $\alpha_{Ind.}$, the individual learning rate. Both the adjusted[3] and non-adjusted[4] pseudo-R^2 is reported. DS means description sensitive.

Type	Nbr	$\alpha_{Obs.}^{Best}$	$\alpha_{Obs.}^{Worst}$	κ^{Best}	κ^{Worst}	DS	# param.	$\overline{AIC}$	Adj. ρ^2	ρ^2
Individual learning	1	-	-	-	-	No	2	75.75	0.129	0.175
Pure observational outcome learning	2	X	-	-	-	Yes	3	66.09	0.240	0.309
	3	X	X	-	-	Yes	4	59.01	0.322	0.414
	4	X		-	-	No	3	58.77	0.325	0.394
	5	-	X	-	-	Yes	3	68.41	0.214	0.283
	6	$\alpha_{Ind.}$	-	-	-	Yes	2	67.16	0.228	0.274
	7	$\alpha_{Ind.}$	X	-	-	Yes	3	59.43	0.317	0.386
	8	-	$\alpha_{Ind.}$	-	-	Yes	2	69.09	0.206	0.252
	9	X	$\alpha_{Ind.}$	-	-	Yes	3	58.76	0.325	0.394
	10	$\alpha_{Ind.}$		-	-	No	2	58.50	0.328	0.374
Pure copying	11	-	-	X	-	Yes	3	75.95	0.127	0.196
	12	-	-	X	X	Yes	4	75.77	0.129	0.221
	13	-	-	-	X	Yes	3	75.62	0.131	0.200
	14	-	-	X		No	3	75.43	0.133	0.202
Mixed strategy learning (observational outcome learning + copying)	15	X	-	X	-	Yes	4	66.21	0.239	0.331
	16	X	-	X	X	Yes	5	65.98	0.242	0.357
	17	X	-	-	X	Yes	4	65.88	0.243	0.335
	18	X	-	X		Yes	4	65.54	0.247	0.339
	19	X	X	X	-	Yes	5	59.20	0.320	0.435
	20	X	X	X	X	Yes	6	58.66	0.326	0.464
	21	X	X	-	X	Yes	5	58.57	0.327	0.442
	22	X	X	X		Yes	5	58.42	0.329	0.443
	23	X		X	-	Yes	4	59.19	0.320	0.412
	24	X		X	X	Yes	5	58.57	0.327	0.442
	25	X		-	X	Yes	4	58.29	0.330	0.422
	26	X		X		No	4	58.36	0.329	0.421
	27	-	X	X	-	Yes	4	68.67	0.211	0.303
	28	-	X	X	X	Yes	5	68.37	0.214	0.329
	29	-	X	-	X	Yes	4	68.01	0.218	0.310
	30	-	X	X		Yes	4	68.12	0.217	0.309
	31	$\alpha_{Ind.}$	-	X	-	Yes	3	67.70	0.222	0.291
	32	$\alpha_{Ind.}$	-	X	X	Yes	4	67.37	0.226	0.318
	33	$\alpha_{Ind.}$	-	-	X	Yes	3	67.00	0.230	0.299
	34	$\alpha_{Ind.}$	-	X		Yes	3	66.84	0.232	0.301
	35	$\alpha_{Ind.}$	X	X	-	Yes	4	59.98	0.311	0.403
	36	$\alpha_{Ind.}$	X	X	X	Yes	5	59.28	0.319	0.434
	37	$\alpha_{Ind.}$	X	-	X	Yes	4	59.06	0.321	0.413
	38	$\alpha_{Ind.}$	X	X		Yes	4	59.18	0.320	0.412
	39	-	$\alpha_{Ind.}$	X	-	Yes	3	69.09	0.206	0.275
	40	-	$\alpha_{Ind.}$	X	X	Yes	4	68.96	0.207	0.299
	41	-	$\alpha_{Ind.}$	-	X	Yes	3	69.04	0.206	0.275
	42	-	$\alpha_{Ind.}$	X		Yes	3	68.89	0.208	0.277
	43	X	$\alpha_{Ind.}$	X	-	Yes	4	58.97	0.322	0.414
	44	X	$\alpha_{Ind.}$	X	X	Yes	5	58.52	0.327	0.442
	45	X	$\alpha_{Ind.}$	-	X	Yes	4	58.57	0.327	0.419
	46	X	$\alpha_{Ind.}$	X		Yes	4	58.27	0.330	0.422
	47	$\alpha_{Ind.}$		X	-	Yes	3	59.10	0.321	0.390
	48	$\alpha_{Ind.}$		X	X	Yes	4	58.57	0.327	0.419
	49	$\alpha_{Ind.}$		-	X	Yes	3	58.24	0.331	0.400
	50	$\alpha_{Ind.}$		X		No	3	58.26	0.330	0.399

The best overall model corresponds to model 49 in Supplementary Table 2. For those participants whose behavior was best captured by a pure observational outcome learning model, the best model corresponds to model 3. For those participants whose behavior was best captured by a mixed strategy learning model, the best model corresponds to model 48. In our model-based fMRI-analyses, for practical reasons we used the full (maximum) model, i.e. model number 20 in the table above. From this model, we used the copying rate, κ. The mean copying rate for demonstrators described as Best was -0.04 (sd = 0.34), and for demonstrators described as Worst -0.23 (sd = 0.44), see Supplementary Fig. 4.

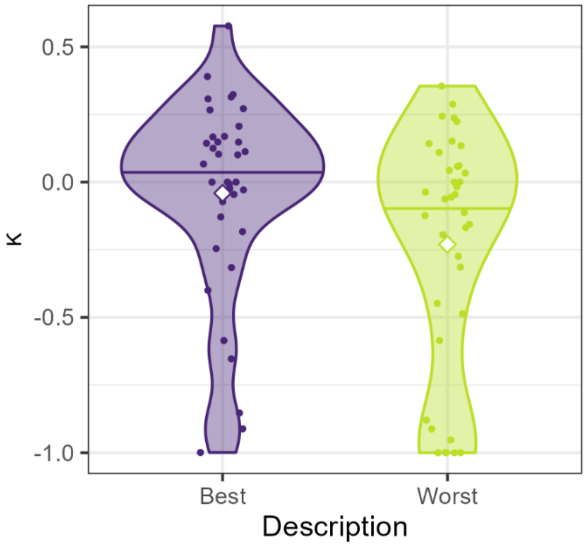

Supplementary Figure 4. Copying rate.
Distribution of the copying rate κ for the full (maximum) model.

Post-experiment verbal reports

To verify that our manipulation was successful we asked participants in a funneled debriefing if they believed that the demonstrators had been real previous participants. Of the 38 participants, 26 reported that they believed the information, 6 reported that they did not believe it and 5 reported that they partially believed it (for instance that they believed the information in the beginning of the paradigm but not towards the end). Data was missing for one participant.

We further wanted to see if the categorization of participants' learning strategies based on RL modeling had some overlap with the categorization based on how the participants themselves said that they used observational information post-experiment. With regards to learning by combining observed choice with observed outcome, 32 was categorized as learning in this manner, 5 was categorized as not. Roughly half, 17 participants, were categorized as copying or avoiding the choice made by the demonstrators, while 20 were not. And finally, 18 participants were categorized as being description-sensitive from how they reported how they learned, while 19 were not. Data from the debriefing was missing for one participant. For those that stated that they used some form of observational outcome learning, 93.75% were best described by a model including observational outcome learning. For those that stated that they to some extent copied (or avoided) choices made by the demonstrators, 70.59% were best described by a model that included copying. For those that stated that they to some extent used observational information differently as a function of the demonstrator's description, 72.22% were best described by a model that included some form of description-sensitive learning. Although we did not necessarily expect verbal report to coincide with modeling results, the existing overlap still gives some validation to our modeling results.

Additional analyses:

Ratings

Participants rated how big of an advantage on a scale from 1 ("No advantage") to 5 ("Big advantage") they felt that it had been to observe the demonstrators. Mean rated advantage was 3.81, the median rated advantage was 4.

Participants own mean rated their performance was 3.35 (sd = 0.82). Two one-tailed t-tests showed that demonstrators described as best were rated higher but not significantly so ($p = 0.34$; mean rating difference between own and demonstrator rating = 0.16, sd = 1.01), while demonstrators described as worst were significantly rated as having lower performance ($t(36) = 4.4$, $p < 0.001$; mean rating difference between own and demonstrator rating = 0.65, sd = 0.89).

Effect of block difficulty on performance

We further tested if performance was affected by block difficulty, i.e. the difference in probability of receiving shocks given an optimal compared to suboptimal choice. This was done using a GLMM with the participants' choices coded according to the choice optimality (Optimal, Suboptimal) as the dependent variable, and Description, Trial and Difference as predictors. Again, we saw an effect of Trial ($p = 0.003$), indicative of learning over time. There was also an effect of Difference ($p = 0.0001$), such that participants performed better during easier blocks where the difference in probability of receiving a shock between optimal compared to suboptimal choices was larger. Again, there was no significant effect of Description on choice optimality ($p = 0.996$). See Supplementary Figure 5.

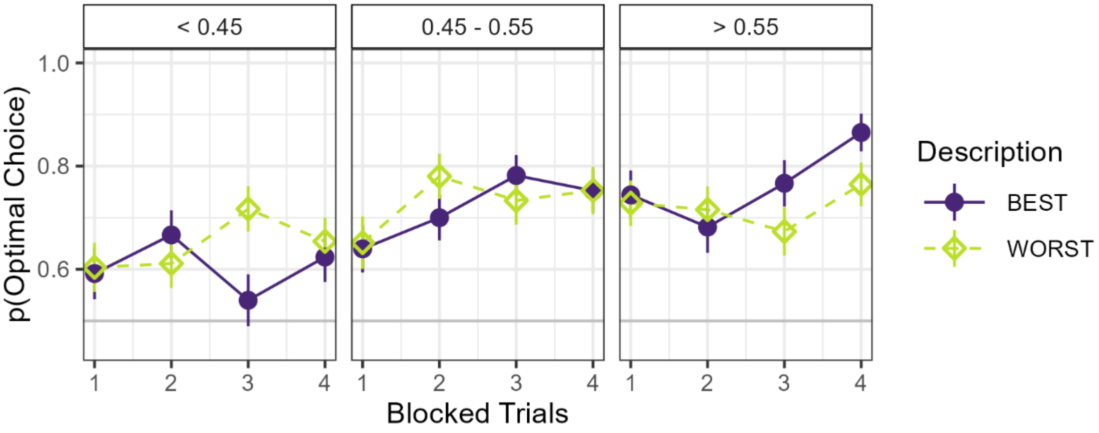

Supplementary Figure 5. Effect of difficulty on performance.
Performance as an effect of Description, Trial and Difference. Data are separated into three subsets depending on the size of the difference in probability to receive a shock after selecting the optimal compared to suboptimal choice. Trials are blocked pairwise for clarity.

Reaction time

To investigate possible explanations for our results we also investigated the effect of Description or strategy use (i.e. whether or not the participant belonged to the subgroup of participants that were described as observational outcome learners or those described as mixed strategy learners that also copied) on reaction time, RT. Analyses were carried out using a series of GLMMs with RT as the dependent variable. Here, we used the inverse gaussian distribution together with an identity link function.

Our analyses showed no differences in RT as a function of either Description ($p = 0.51$) or strategy use ($p = 0.23$). Neither was there any significant interaction between Description and strategy use ($p = 0.62$).

How well do the models capture behavior over time

To investigate how well the models of interest captured behavior over time we plotted the predicted behavior given the models against actual behavior, Supplementary Figure 5, as well the probability of the data over time for each of the three models of interest, Supplementary Figure 6. All three models appear to capture behavior over time relatively well, with no clear difference in the models' predictive abilities over time. All models appear to predict behavior slightly better towards the end of a block and possibly also at the very beginning.

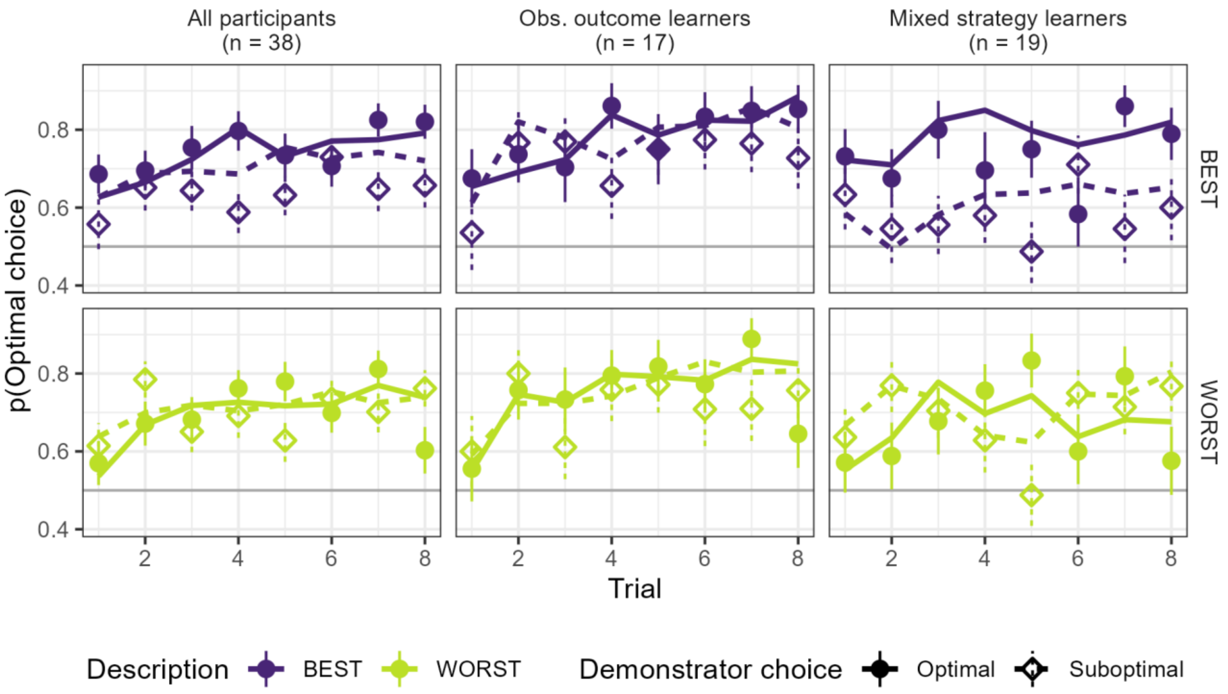

Supplementary Figure 6. Model predictions over time.

Model based predicted mean behavior (lines) plotted against actual mean behavior (points) over time, for the three RL models that best describe either all participants or the two main subgroups of participants best described by either observational outcome learning models or mixed strategy learning models. Error bars indicate standard error of the mean.

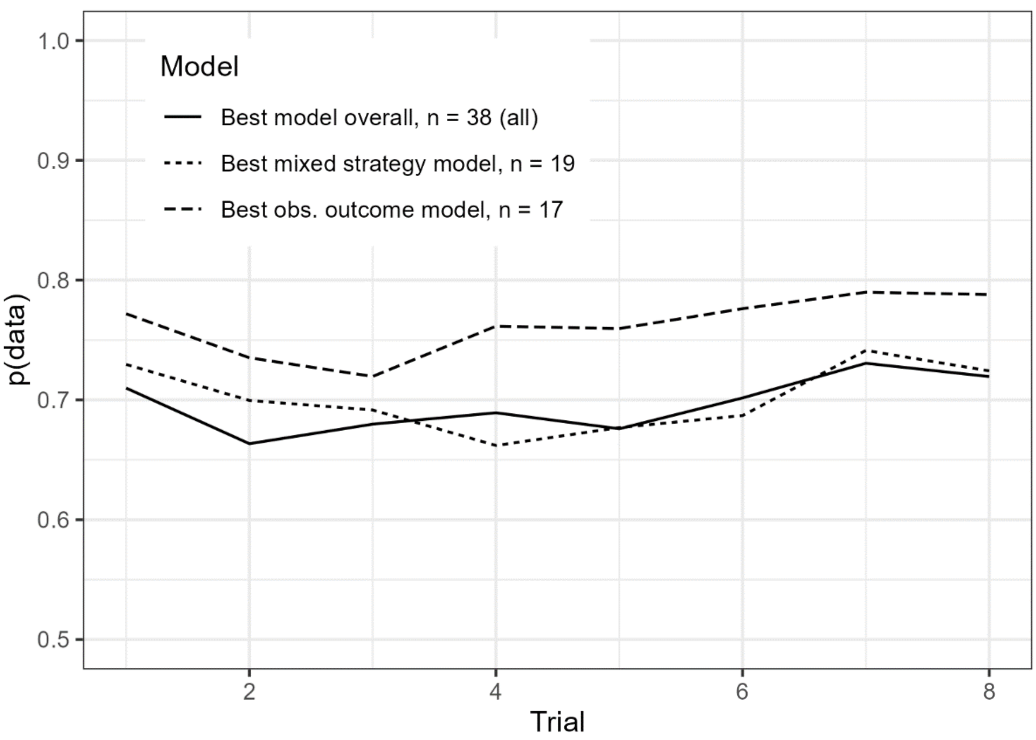

Supplementary Figure 7. Probability of the data.

Trial-by-trial measures of the probability of the data (participants' behavior) for each of the three models of interest.

Comparing the present study to the previous

In an attempt to understand the differences in behavior between the present study and the previous study[1], which the present one is based on, we compared performance between them. To do this, we compared the data in the previous study where the observed demonstrators performed poorly and had actual low ability, but removed the data where demonstrators performed well and had actual high ability. The difference in performance was compared separately for each level of described ability, while also taking the difficulty of each block into account. The analyses were carried out using GLMMs, where the dependent variable was the participants' choices coded according to the choice optimality (Optimal, Suboptimal). As predictors we included Study (Present coded as 0, Previous coded as 1) and Difficulty. For the analysis of the subset of the data where participants observed a demonstrator described as high in ability/Best, there was a main effect of Study ($\beta = 0.45$, $SE = 0.20$, $Z = 2.21$, $p = 0.027$), such that performance was worse in the present study. For the analysis of the subset of the data where participants observed a demonstrator described as low in ability/Worst, there was no effect of Study ($p = 0.607$), performance did not differ between the studies, see Supplementary Figure 8.

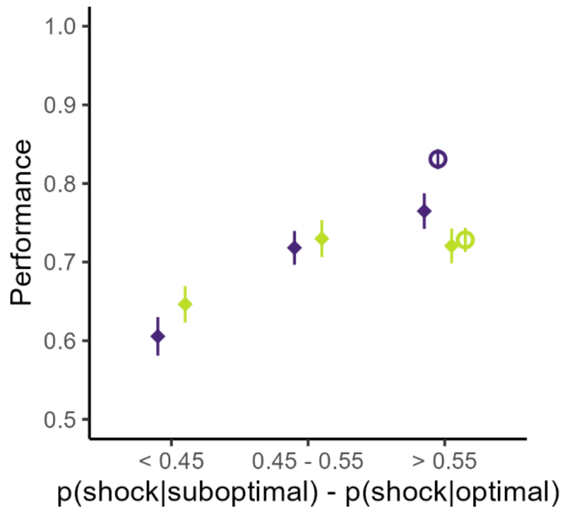

Supplementary Figure 8. Performance in the previous and present study plotted against each other. Data is plotted over three ranges of differences in the probability of shock given choice (suboptimal, optimal) to include effects of the difficulty of the block. Note that the difference in probability was fixed at 0.6 in the previous study.

What then drives this difference in performance between the studies? To try to answer this we also compared performance in the previous study with performance of the two subsets of participants identified in the present study, again separately for each level of described ability. There was no difference in performance between the observational outcome learners and participants in the previous study (High ability/Best: p = 0.759; Low ability/Worst: p = 0.070). However, mixed strategy learners did perform worse than participants in the previous study when demonstrators were described as high in ability/Best ($\beta = 0.58$, $SE = 0.26$, $Z = 2.51$, $p = 0.024$), but not when demonstrators were described as low in ability/Worst (p = 0.240).

Our analyses (although based on a small sample) thus suggest that even though almost half (N = 17) of our participants seem to learn the task in a similar manner as participants in the previous study, an almost equally sized group (N = 19) in the present study instead copies to a larger extent and performs worse.

Supplementary References

1. Selbing, I. & Olsson, A. Beliefs about Others' Abilities Alter Learning from Observation. *Sci. Rep.* **7**, 16173 (2017).

2. Wilson, R. C. & Collins, A. G. E. Ten simple rules for the computational modeling of behavioral data. 1–35 (2019).

3. Camerer, C. & Ho, T.-H. Experience-weighted Attraction Learning in Normal Form Games. *Econometrica* **67**, 827–874 (1999).

4. McFadden, D. Conditional logit analysis of qualitative choice behavior. in *Frontiers in econometrics* 104–142 (Academic Press, New York, 1974).
